# Correlation between skin temperature in the lower limbs and biochemical marker, performance data, and clinical recovery scales

**DOI:** 10.1371/journal.pone.0248653

**Published:** 2021-03-18

**Authors:** Gabriela de Carvalho, Carlos Eduardo Girasol, Luiz Guilherme Cruz Gonçalves, Elaine Caldeira Oliveira Guirro, Rinaldo Roberto de Jesus Guirro

**Affiliations:** 1 Postgraduate Program in Rehabilitation and Functional Performance, Department of Healthy Science, Ribeirão Preto Medical School, University of São Paulo, Ribeirão Preto, São Paulo, Brazil; 2 Physiology department of Botafogo Futebol Clube, Ribeirão Preto, São Paulo, Brazil; Universidade Federal de Juiz de Fora, BRAZIL

## Abstract

The aim of this study was to evaluate the correlation between tools commonly used in the detection of physiological changes, such as clinical complaints, a biochemical marker of muscle injury, and performance data during official matches, with infrared thermography, which has been commonly used in the possible tracking of musculoskeletal injuries in athletes. Twenty-two athletes from a professional soccer club (age 27.7 ± 3.93 years; BMI 24.35 ± 1.80 kg/cm^2^) were followed during the season of a national championship, totaling 19 matches with an interval of 7 days between matches. At each match, the athletes used a Global Positioning System (GPS) device to collect performance data. Forty-eight hours after each match, every athlete’s perception of recovery, fatigue, and pain was documented. Blood was collected for creatine kinase (CK) analysis, and infrared thermography was applied. Only athletes who presented pain above 4 in either limb were included for thermographic analysis. Each thermographic image was divided into 14 regions of interest. For statistical analysis, we included only the images that showed differences ≥ 1° C. Data normality was verified by the Kolmogorov-Smirnov test with Dallal-Wilkinson-Lilliefors correction. We used the Pearson correlation coefficient to verify the correlation between infrared thermography and the biochemical marker, performance data, and clinical recovery scales. No correlation was observed between mean skin temperature and blood CK levels, pain level, perception of recovery, and fatigue perception (r <0.2, p>0.05). Thus, infrared thermography did not correlate with CK level, pain, fatigue perception, or recovery, nor with performance variables within the field.

## Introduction

Professional soccer is considered a sport with a high risk of injury due to a constant combination of physical and psychological stress [1]. These injuries are related to multifactorial causes and may be associated with previous injuries, flexibility, strength, fatigue, central stability, biomechanics, and workload [2]. Reported injuries in soccer occur more frequently in the lower limbs, being the thigh the most affected area (41.9% of the total), followed by the knee (19.0%), most of which occur in muscle injury conditions. Regarding severity, 49.3% were considered moderate and 48.5% severe. There is a high injury rate present in soccer, with muscle injuries accounting for 37% of all time away from playing due to injuries in professional soccer; the four largest muscle groups of the lower limbs correspond to 90% of these injuries [3].

There is a constant exposure of this population to a series of physical overloads through training and matches that cause physiological changes such as fatigue, increased concentrations of muscle-damaging and pro-inflammatory enzymes, and subjective changes such as pain [4–6], as well as increased blood flow. Through vasodilation and the increase in the volume of blood circulating in the region, it is possible to observe skin temperature influence [7].

The tracking of post-exercise recovery is directly linked to injury prevention [8]. The common use of techniques and tools is necessary for a better recovery and, consequently, preventing the risk of injury. The interaction among metabolic tools to detect increased biochemical markers, such as creatine kinase (CK), performance analysis through physical tests, and the athlete’s subjective perception of physical effort and pain, is essential in the athlete’s follow-up routine [9, 10].

Recently, Infrared Thermography (IRT) has been implemented in the sports scene because it is a non-invasive, painless method that does not require contact with the body region to be evaluated. It is based on the emission of infrared radiation by bodies with a temperature above absolute zero, providing an image of the corporal skin temperature distribution [11], which is conditioned by microcirculatory, metabolic, and autonomic activities [12, 13]. It is possible to monitor in real-time the physiological functions related to the control of skin temperature [14–16]. The literature shows that asymmetries, equal or above 1°C between contralateral limbs, can indirectly indicate–through an installed inflammatory process–a potential risk of injuries related to training and competition load [7, 17, 18] and that early detection of different temperature values between the contralateral limbs, followed by an injury prevention protocol, can significantly reduce the number of injuries during the season [17, 18]. However, there is still little scientific evidence and conflicting results regarding its applicability within different sports scenarios in the literature [19, 20].

The knowledge about the correlation between increased skin temperature and asymmetry between the limbs that IRT can detect, with the instruments used to track physiological changes, is still restricted to possible use in clinical practice in high-performance sports. Considering that, in the presence of a muscle injury, there is an installation of an inflammatory process, generating an increase in blood flow and consequently a rise in local temperature, an increase in pain and muscle damage enzymes, it is expected that there is an interaction between the tools that can evaluate these parameters. The study aimed to assess the correlation between thermography and commonly used tools, such as common clinical complaints in athletes, a biochemical marker for muscle damage, and performance data during official matches.

## Materials and methods

### Ethics

The study was approved by the Ethics Committee on Research in Human Beings of the Institution Ethics Committee (protocol 3.130.372). Consent was obtained in the form of a written document. Throughout the data collection process, the athletes and the technical commission received access to all the photographs captured and the feedback from the team of professionals involved in the research regarding the individual evaluation of each athlete.

### Study design

This is a single-blind cross-sectional study conducted at a professional soccer club during the season of a national championship (2018). All athletes from the club were invited to participate, for whom procedures were clarified, and those who agreed signed an informed consent form.

**Fig 1:**
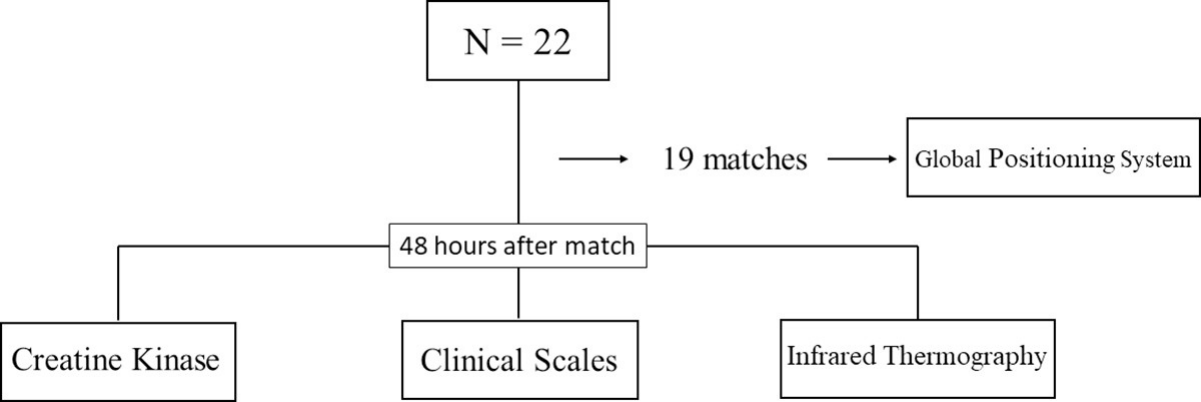
Flowchart of the analyses.

The athletes were followed in 19 matches, with an interval of 7 days between matches. At each match, the athletes used a Global Positioning System (GPS) device to collect performance data. Forty-eight hours after each match, every athlete’s perception of recovery, fatigue, and pain was documented, blood was collected for creatine kinase analysis, and infrared thermography was applied. A flowchart explaining the sequence of analyses can be seen in Fig 1.

### Sample

The sample size calculation processing was performed using the software Ene version 3.0 (Autonomous University of Barcelona, Barcelona, Spain). The calculation was made to determine a correlation of 0.5, according to Zou *et al*. (2003), for a moderate correlation. Thus, 22 participants were estimated to reach a statistical power of 80% and an α of 0.05 [21].

Participants were healthy male athletes between 18 to 35 years, soccer professionals of the same club, competing in the official national championship and the state championship. They were followed for five months during the national C series championship, totaling 19 matches and 19 weeks of follow-up. They should present pain of at least 4 [22] according to the visual analog scale in any region of the lower limbs and should have played at least 72 minutes of the match–more than 60% of the match with a sufficient workload for GPS analysis–prior to data collection to be included in the study. Forty-eight hours after each match, evaluations were carried out with the players. The evaluations were conducted in this period because the peak of muscle pain and creatine kinase present in the blood is between 48 and 72 hours after exercise. All data related to the perception of recovery, fatigue, pain, a blood sample for CK analysis, and thermographic photos were collected. In all matches, the GPS data of each player was collected to assess their performance during the match. Data related to the performance of these participants were also collected by the technical committee, as YOYO IR—Intermittent Recovery Test Level 1 and Countermovement Jump (CMJ), to detail the profile of these athletes. The exclusion criteria were the use of illicit, anabolic, anti-inflammatory, or analgesic drugs, alcohol intake, or the presence of lesions at the beginning of the study.

### Infrared thermography

Three infrared images were captured from the lower limbs in both the anterior and posterior views, at a distance of 100cm from each volunteer, to allow the limbs’ best framing. All photographs were captured 48 hours after matches [18]. The athletes were instructed two hours before the picture not to consume caffeine, chocolate, nicotine, or alcohol and not to use ointments or creams [23, 24].

To capture the images, the athletes remained in the orthostatic position for 15 minutes in a temperature-controlled environment at 23±2°C [25] with fluorescent lighting, without direct irradiation by sunlight or heat or cold generators, and with the segments to be photographed free of any clothing [26].

**Fig 2:**
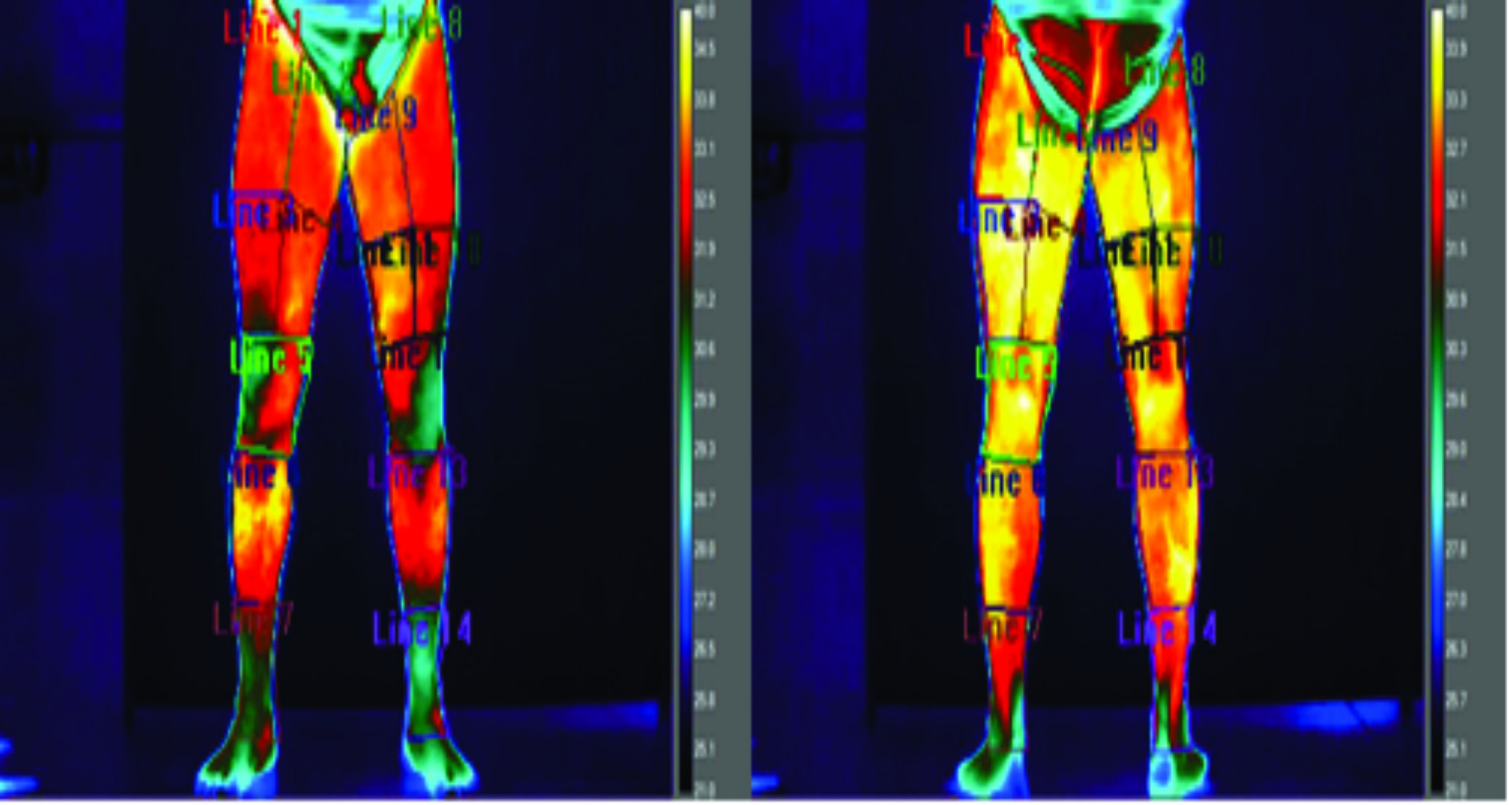
Infrared thermography with 14 ROIs.

A T450 model thermal camera (FLIR^®^ Systems, Danderyd, Sweden) was used, with a precision of up to 0.05° Celsius (C) and emissivity of 0.98 [27]. The images were analyzed using QuickReport software version 1.2 (FLIR^®^ Systems) by two previously trained physiotherapists. Each leg was divided into seven regions of interest (ROI), totaling 14 anterior ROIs and 14 posterior ROIs (Fig 2) to analyze each member region specifically. All images were analyzed with the same color palette and in the fixed range of 21-40°C.

The mean temperature asymmetries between corresponding ROIs in the contralateral limbs were analyzed. When one or more asymmetries were observed, and there was a report of pain in one region, the athlete was included for the subsequent correlations with the other variables. The mean temperature asymmetries were considered when the athlete presented a difference of 1°C between the limbs [18].

### Creatine kinase

Forty-eight hours after the match, 32 μl of capillary blood was collected from each athlete’s digital pulp and deposited on a CK reactive strip for analysis in the Reflotron Plus System (Roche Diagnostics International, Rotkreuz, Switzerland) [28].

### Global positioning system

A 15Hz GPS unit (SPI HPU, GPSports, Canberra, Australia), (CV = 0.9%, SEM = 0.07) [29] was attached to each player’s shorts before the matches. All players used the same equipment during the competition period. Measurements obtained corresponded to the total distance covered (TD), measured in meters, High-intensity distance covered (HID)–total distance covered at a velocity >5.5 m.s^-1^, the total number of Acceleration (high intensity) (TNA)–acceleration >3 m.s^-1^ for a period longer than 0.5 s, the total number of Deceleration (high intensity) (TND)—deceleration >3 m.s^-1^ for a period longer than 0.5 s, the total number of sprints (TNS)–velocity >5.5 m.s^-1^ for at least 1s and maintained at a velocity greater than 4.4 m.s-^1^ [30–32].

### Clinical recovery scales

Throughout the season, scales were applied to assess recovery perception, pain perception, fatigue perception. All athletes have been previously introduced to such scales one month before the beginning of the study, using them at least once a week.

Forty-eight hours after the last match, with the other assessments cited, the perception of recovery was measured on a 10-point Likert scale, and each athlete was asked in the same way: “From 1 to 10, how recovered are you feeling today? where ‘1’ indicates “not recovered” up to ‘10’ as “fully recovered” [33], as well as fatigue, “From 1 to 10, how tired are you now?” where ‘1’ means “not fatigued at all” and ‘10’ means “extremely fatigued” [34]. The subjective pain score was obtained using the visual analog scale, graded from ‘0’ to ‘10’, where each athlete was asked in a standard way: “From 0 to 10, how much pain are you feeling today?” where ‘0’ stands for the total absence of pain and ‘10’ the maximum level of pain that can be endured”, as described by Sellwood *et al*. [35] and Parouty *et al*. [36]. In the presence of punctual pain in any region of the lower limbs, the athlete reported the location of the pain. Still, they must have experienced at least pain 4 based on the visual analog scale for inclusion in the study and comparison with thermographic images.

### Statistical analysis

The Kolmogorov-Smirnov test with Dallal-Wilkson-Liliefours correction verified data normality. The Pearson correlation coefficient was used to verify the correlation between infrared thermography and the following variables: creatine kinase, perception scales recovery, fatigue, and pain, in addition to field activities by GPS.

The magnitude of the correlations was interpreted by the classification established by Munro [37]: low, from 0.26 to 0.49; moderate, from 0.50 to 0.69; high, from 0.70 to 0.89; very high, from 0.90 to 1.00. All processing was performed using Prism^®^ software, version 7.00 (San Diego, California, USA).

## Results

A total of 22 athletes (age 27.7 ± 3.93 years; BMI 24.35 ± 1.80 kg/cm^2^) participated in the study, and Table 1 shows the athlete’s anthropometric and performance characteristics. One hundred fifty-nine infrared images were analyzed, of which only 56 were used for correlation analysis, as they presented asymmetry regarding the temperature of the contralateral limbs and corresponded with the report of pain in one limb. The mean and standard deviation of the asymmetry values between the limbs, clinical variables scales, and the GPS values of the individuals included in the study in all the matches are presented in Table 2.

**Table 1:** Description of the mean values and standard deviations of the anthropometric data, performance tests (YOYO IR—Intermittent Recovery Test Level 1 and Countermovement Jump), clinical variables, and the GPS values of the individuals included in the study (n = 22).

Anthropometric and Performance Measures	Mean ± SD
Age (years)	27.7 ± 3.93
Height (m)	1.78 ± 0.06
Body Mass (Kg)	77.8 ± 8.65
BMI (Kg/cm^2^)	24.35 ± 1.80
YOYO IR (m)	1718.0 ± 386.2
CMJ (cm)	43.1 ± 3.9

**Table 2:** Description of the mean values and standard deviations of all matches of temperature asymmetry analyzed by IRT, clinical and biochemical variables, and the GPS values of the individuals included in the study (n = 22).

Variables	Mean ± SD
Temperature asymmetry (°C)	1.58 ± 0.84
Creatine Kinase (IU/L)	805.7 ± 560.5
Pain	4.7 ± 1.6
Perception of recovery	3.5 ± 1.1
Perception of fatigue	4.8 ± 1.4
Mean Time in a match (min)	90.16 ± 10.74
Total Distance covered (m)	9017.04 ± 1621.48
High-intensity distance (m)	1140.90 ± 494.61
Total number of high-intensity acceleration (m)	15.60 ± 7.68
Total number of high-intensity deceleration (m)	22.60 ± 8.97

No correlation was observed regarding mean skin temperature analyzed by infrared thermography with blood CK level, pain level, perception of recovery, or fatigue perception. The correlation values (r) between the physiological variables and the global positioning system with a mean temperature of the total area of the left and right lower limb are presented in Table 3, and with the mean values of temperature of the region of interest of the left and right lower limb–anterior (Table 4) and posterior view (Table 5).

**Table 3:** Correlation with mean cutaneous temperature values of the left and right lower limb anterior total area (ATA) and posterior total area (ATP) with physiological variables and Global Positioning System (n = 22).

**Infrared Thermography**	**CK**	**Pain**	**Fatigue**	**Recovery**	**TD**	**HID**	**TNA**	**TND**	**TNS**
**ATA**	r = 0.03	r = -0.18	r = -0.19	r = 0.13	r = -0.10	r = -0.14	r = 0.12	r = 0.23	r = 0.03
**PTA**	r = 0.10	r = -0.13	r = -0.19	r = 0.10	r = -0.08	r = -0.09	r = 0.03	r = 0.19	r = -0.03

ATA—anterior total area; PTA- posterior total area; CK—Creatine kinase; TD–total distance covered; HID–High-intensity distance; TNA–Total number of acceleration (High-intensity); TND—Total number of deceleration (High-intensity).

**Table 4:** Correlation of the average temperature values of the cutaneous regions of interest of the left and right lower limb–anterior view, with physiological variables and Global Positioning System (n = 22).

Anterior View									
Infrared Thermography	CK	Pain	Fatigue	Recovery	TD	HID	TNA	TND	TNS
**ROI 1**	r = -0.06	r = -0.05	r = -0.003	r = -0.06	r = -0.07	r = -0.10	r = 0.12	r = 0.16	r = -0.0008
**ROI 2**	r = -0.09	r = -0.06	r = -0.05	r = 0.05	r = -0.12	r = -0.05	r = 0.19	r = 0.18	r = 0.06
**ROI 3**	r = 0.01	r = -0.04	r = 0.03	r = -0.06	r = -0.07	r = -0.04	r = 0.10	r = 0.22	r = -0.0007
**ROI 4**	r = 0.05	r = -0.08	r = -0.06	r = -0.09	r = -0.17	r = -0.23	r = -0.04	r = -0.01	r = -0.13
**ROI 5**	r = 0.06	r = -0.02	r = -0.14	r = 0.12	r = -0.08	r = -0.07	r = 0.09	r = 0.14	r = -0.01
**ROI 6**	r = 0.07	r = -0.22	r = -0.16	r = 0.02	r = -0.22	r = -0.18	r = -0.04	r = 0.007	r = -0.18
**ROI 7**	r = 0.05	r = -0.15	r = -0.20	r = 0.18	r = -0.09	r = -0.12	r = 0.13	r = 0.20	r = 0.07
**ROI 8**	r = -0.001	r = -0.07	r = -0.04	r = -0.004	r = -0.15	r = -0.16	r = 0.16	r = 0.21	r = 0.07
**ROI 9**	r = -0.08	r = -0.11	r = -0.04	r = -0.01	r = -0.15	r = -0.12	r = 0.11	r = 0.13	r = -0.02
**ROI 10**	r = -0.02	r = -0.20	r = -0.17	r = 0.03	r = -0.10	r = -0.16	r = 0.09	r = 0.17	r = 0.06
**ROI 11**	r = -0.02	r = -0.14	r = -0.10	r = 0.09	r = -0.02	r = -0.12	r = 0.07	r = 0.16	r = -0.01
**ROI 12**	r = 0.13	r = -0.12	r = -0.14	r = 0.09	r = -0.09	r = -0.02	r = 0.22	r = 0.33	r = 0.12
**ROI 13**	r = 0.08	r = -0.02	r = -0.08	r = 0.02	r = -0.13	r = -0.26	r = -0.02	r = 0.12	r = -0.05
**ROI 14**	r = 0.004	r = -0.08	r = -0.003	r = 0.19	r = 0.04	r = -0.02	r = 0.12	r = 0.25	r = 0.07

ROI—region of interest; CK—Creatine kinase; TD–total distance covered; HID–High-intensity distance; TNA–Total number of acceleration (High-intensity); TND—Total number of deceleration (High-intensity).

**Table 5:** Correlation of the average temperature values of the cutaneous regions of interest of the left and right lower limb–posterior view, with physiological variables and Global Positioning System (n = 22).

Posterior View									
Infrared Thermography	CK	Pain	Fatigue	Recovery	TD	HID	TNA	TND	TNS
**ROI 1**	r = -0.10	r = -0.05	r = -0.11	r = -0.10	r = -0.23	r = -0.19	r = 0.03	r = 0.12	r = -0.11
**ROI 2**	r = -0.08	r = -0.13	r = -0.06	r = 0.08	r = -0.10	r = -0.08	r = -0.02	r = 0.08	r = 0.01
**ROI 3**	r = -0.10	r = 0.005	r = -0.03	r = 0.02	r = -0.21	r = -0.06	r = 0.08	r = 0.18	r = -0.04
**ROI 4**	r = -0.10	r = -0.10	r = -0.04	r = -0.01	r = -0.04	r = -0.09	r = -0.02	r = 0.04	r = -0.02
**ROI 5**	r = -0.15	r = -0.01	r = -0.08	r = 0.17	r = -0.20	r = -0.10	r = 0.09	r = 0.08	r = -0.07
**ROI 6**	r = -0.21	r = -0.04	r = -0.006	r = 0.01	r = -0.13	r = -0.09	r = -0.09	r = -0.09	r = -0.24
**ROI 7**	r = -0.30	r = -0.05	r = -0.13	r = 0.08	r = -0.09	r = -0.04	r = 0.08	r = 0.15	r = -0.03
**ROI 8**	r = -0.13	r = -0.13	r = -0.27	r = -0.01	r = -0.20	r = -0.20	r = 0.03	r = 0.03	r = -0.09
**ROI 9**	r = -0.13	r = -0.03	r = -0.06	r = -0.09	r = -0.27	r = -0.27	r = -0.06	r = 0.03	r = -0.17
**ROI 10**	r = -0.18	r = -0.12	r = -0.23	r = 0.02	r = 0.08	r = 0.09	r = 0.18	r = 0.32*	r = 0.17
**ROI 11**	r = -0.08	r = -0.08	r = -0.08	r = 0.05	r = -0.18	r = -0.17	r = -0.03	r = 0.10	r = -0.05
**ROI 12**	r = -0.13	r = 0.01	r = 0.03	r = 0.16	r = 0.01	r = 0.02	r = 0.04	r = 0.18	r = -0.01
**ROI 13**	r = -0.14	r = -0.03	r = -0.08	r = 0.11	r = -0.003	r = -0.03	r = -0.07	r = 0.06	r = -0.13
**ROI 14**	r = -0.12	r = -0.14	r = -0.09	r = 0.11	r = 0.05	r = 0.02	r = 0.12	r = 0.32	r = 0.12

ROI—region of interest; CK—Creatine kinase; TD–total distance covered; HID–High-intensity distance; TNA–Total number of acceleration (High-intensity); TND—Total number of deceleration (High-intensity).

## Discussion

This study aimed to correlate the increase in skin temperature using infrared thermography with other physiological aspects such as pain, increased CK, feeling of fatigue, and performance during soccer matches. Our main findings show no correlation between the increase in skin temperature and the presence of pain in the lower limbs and the increase in CK, feeling of fatigue, and the GPS variables.

Thermography has been used regularly in the routine of soccer club evaluations as a tool for the prevention of muscle injuries, generally only due to the assessment of differences in skin temperature in areas of interest [14]. Bandeira *et al*. [38] also monitored the professional soccer club but evaluated the minimization of injury risk. Their findings point out that thermography can be appointed together with CK. However, Da Silva *et al*. [39] correlated CK and thermographic images, and it was observed that there was no correlation between the two variables after a muscle injury induction protocol, so it can be said that in a real situation–matches and training diaries–like the one investigated in our study, and laboratory situations, it is not possible to see a correlation between biomarkers of muscle damage with the increase in skin temperature.

It is known that the peak of delayed-onset muscle soreness (DOMS), as well as the increase in creatine kinase, are between 48 and 72 hours after physical activity [28, 40]. Studies in the literature use the 48-hour window [18, 39] after a physical effort to apply IRT, following the same window of CK responses and DOMS. Nevertheless, as mentioned before, it was not possible to observe a correlation among these variables in this window between physical effort and evaluation. The ideal moment for the application of IRT seems to be of extreme importance in the assessment results. From our results, it appears that a better evaluation is necessary for the application window of such a tool.

Other factors were considered in relation to our study. We can say that the athlete’s positioning and the specificity of each one’s gestures during the match can exert influences on different muscle groups [41]. Thus, our study had a rigorous screening process for including images for correlation analysis. Unlike other thermographic studies [14, 18, 20], this study analyzed 14 ROIs in the anterior and 14 posterior regions, thus ensuring that no muscle could be excluded from the assessment since, in the soccer scenario, the incidence of injuries is located in more significant quantities in muscles of the lower limb [3].

When it comes to injury prevention, a complex approach with different focuses should be used [42]. Therefore, thermography should not replace other assessments and exams but should complement the assessments and support decisions [43]. Our study results contribute further to the relationship between the applicability of infrared thermography and muscle injuries. In contrast, a single tool could not relate to other assessments–subjective, biochemical, and physiological. On the other hand, the excessive workload causes a predisposition to injuries [2], so that thermography can also help map the temperature to the athlete’s workload. However, this assessment timing seems to be crucial, as, at 48 hours of the last match in which field development information was collected, no correlations were observed with the increase in skin temperature. Further studies should be carried out to evaluate the best moment of thermography application in sport, carrying out a systematic implementation of this evaluation during a pre- and post-match period. Perhaps better results can be presented, with the application of infrared thermography immediately before the match, 1 hour, 24, 48, and 72 hours post-match.

The authors did not have access during the collection period to medical records and mapping injuries from the same championship of previous years. Therefore, it was not possible to observe whether the thermographic tracking during the championship was able to reduce the number of injured athletes. The medical resources of the country where the study was carried out are also restricted, so there was no possibility to make a comparison between the thermographic images and gold standard imaging exams, such as ultrasound and magnetic resonance. The ideal moment of application is not defined in the literature yet, and it may be an important bias concerning the results presented. These issues become limitations in the exposure of greater results related to the applicability of infrared thermography in sports. The results of this study show that caution should be taken regarding the use of infrared thermography, the correlation of a method for injury prevention, and the best timing for application.

## Conclusion

Infrared thermography of lower limbs of professional soccer players did not correlate with CK level, pain, fatigue perception, or recovery, nor with performance variables within the field.
